# Evaluation of hypoglycemic effect of *Morus alba* in an animal model

**DOI:** 10.4103/0253-7613.40483

**Published:** 2008

**Authors:** Jamshid Mohammadi, Prakash R. Naik

**Affiliations:** Endocrinology Laboratory, Department of Zoology, University of Mysore, Mysore - 570 006, India

**Keywords:** Cholesterol, diabetes, glucose, insulin, *Morus alba*, streptozotocin, triglyceride

## Abstract

**Objective:**

The objective of the present investigation was to evaluate the therapeutic efficacy of mulberry leaves in an animal model of diabetes.

**Materials and Methods:**

Animals were treated with mulberry leaf extract 400 mg and 600 mg/kg body weight for 35 days. Blood glucose, glycosylated hemoglobin, triglyceride, LDL, VLDL, HDL, blood urea, cholesterol, number of β cells, and diameter of the islets of Langerhans were measured at the beginning and at the end of the experiment.

**Results:**

Blood glucose level and other parameters (except HDL) were elevated in the diabetic group, but were brought to control group level in the diabetic group treated with 600 mg/kg body weight of mulberry leaf extract. The diameter of the islets and the number of β cells were reduced in the diabetic group; both parameters were brought to control group level after treatment with mulberry leaf extract.

**Conclusion:**

Mulberry leaf extract, at a dose of 600 mg/kg body weight, has therapeutic effects in diabetes-induced Wistar rats and can restore the diminished β cell numbers.

Diabetes mellitus is a chronic disease characterized by elevated blood glucose levels and disturbances in carbohydrate, fat, and protein metabolism. These metabolic abnormalities result, in part, from a deficiency of the blood sugar-lowering hormone insulin; this deficiency in insulin results in type 1 diabetes or insulin-dependent diabetes mellitus (IDDM). Type 2 diabetes or non-insulin-dependant diabetes mellitus (NIDDM) is a result of hyperglycemia caused by overproduction of glucose at the hepatic level or because of abnormal β cell function or insulin resistance at target cells.[1]

The chronic hyperglycemia of diabetes is associated with damage, dysfunction, and failure of various organs over the long term.[2] In diabetic rats, the impaired utilization of carbohydrate leads to accelerated lipolysis, resulting in hyperlipidemia.[3,4] Despite the availability of many antidiabetic medicines in the market, diabetes and its related complications continue to be major medical problems. Plant derivatives with purported hypoglycemic properties are used in folk medicine and traditional healing systems around the world. The antihyperglycemic effects of these plants are attributed to their ability to increase insulin output from the pancreas, or inhibit intestinal absorption of glucose, or some other processes.[5]

Many pharmaceuticals used in modern medicine are also of natural, plant origin. There is little information available regarding the efficacy and safety of the herbs used in diabetes.[6] In spite of this the use of herbal remedies continues to increase.

The use of herbal remedies has increased many fold from 1990 onwards in the USA.[7] Substantial efforts have been made in recent years to identify new natural and synthetic antidiabetics. The search for more effective and safer hypoglycemic agents continues to be an important area of research. Andallu et al.[8] and Andallu and Varadacharyulu[9,10] have reported many different medicinal properties of mulberry leaves (*Morus alba*); it is used as an antiphlogistic, diuretic, expectorant, and antidiabetic in traditional Chinese medicine.[11,12]

The objective of the present investigation was to evaluate the therapeutic efficacy of *M. alba* (mulberry) leaves in a diabetes induced model in Wistar rats.

## Materials and Methods

The experimental animals, Wistar rats, were procured from the animal house of the Zoology Department, University of Mysore. The experiment protocol was approved by the Departmental Ethics Committee. The animals were maintained under standard conditions of temperature (20 ± 5°C), with a regular 12-h light/12-h dark cycle. They were allowed free access to standard laboratory food and water *ad libitum* throughout the experiment.

The mulberry leaves were collected from the garden of the Sericulture Department of the University of Mysore. The fourth and fifth leaves from the apex of healthy plants were plucked, washed thoroughly under running tap water, shade dried for 5 days, and ground to a fine powder in an electric mixer. The powdered plant material (850 g) was extracted twice (24 h each time) with 90% ethanol at room temperature. Extracts were filtered with Whatman filter paper No. 1. The filtrate was evaporated until dry, using a Soxhlet evaporator, to obtain 93.5 g of the extract.

The animals, irrespective of sex, with body weight ranging between 150 to 200 g, were distributed into five groups (with eight animals in each group) as follows: (I) control group, (II) control group with mulberry leaf extract treatment, (III) diabetic control group, (IV) diabetic group treated with 400 mg/kg/day of mulberry leaf extract, and (V) diabetic group treated with 600 mg/kg/day of mulberry leaf extract.

Animals of groups III, IV, and V were rendered diabetic by a single intraperitoneal (i.p.) injection of 60 mg/kg of streptozotocin (STZ) freshly prepared in 0.1 M of citrate buffer (pH 4.5). Group I and II animals were injected with buffer alone. After 72 h, blood was drawn from the tail of conscious rats and the glucose content was estimated with a glucometer; blood glucose was estimated every week until autopsy. Ten days after the STZ injection, animals of group II and IV received 400 mg/kg/day, and group V received 600 mg/kg/day, of mulberry leaf extract orally for 5 weeks. Body weight was recorded weekly in every group. After 5 weeks, the animals were fasted overnight and autopsied under light ether anesthesia. Blood was collected in 5% EDTA vials by superior and inferior vena cava punctures for measurement of the biochemical parameters.

Pancreatic tissue was taken from all groups of animals; It was washed, fixed in Bouin-Hollande and dehydrated with alcohol in Bouin-Hollande for 18-20 h. Serial sections of 5-μm thickness were cut using a microtome and every fifth slide was stained using chrome alum hematoxylin and phloxine (CHP) method. The serial sections were observed under a light microscope.[13] One hundred (100) islets were measured from 100 randomly selected cross-sections of the pancreas from each rat; the β cells were also counted.

Plasma glucose was estimated by Trinder's method[14] using a GOD/POD kit. Glycosylated hemoglobin was determined according to the ion exchange resin method.[15] Triglycerides were measured by enzyme-colorimetric method.[16] HDL-cholesterol was assayed by the method of Burstein *et al.*[17] LDL-cholesterol and VLDL-cholesterol was measured by using the formula of Friendwald *et al*.[18] Blood urea was estimated by urea-glutamate dehydrogenase (GLDH) method.

The data was represented as mean ± standard error (SE) and the results were analyzed using analysis of variance (ANOVA). Wherever the variance values were found to be significant at the 5% level, Duncan's multiple range test (DMRT) was applied.

## Results

**Figure 1:**
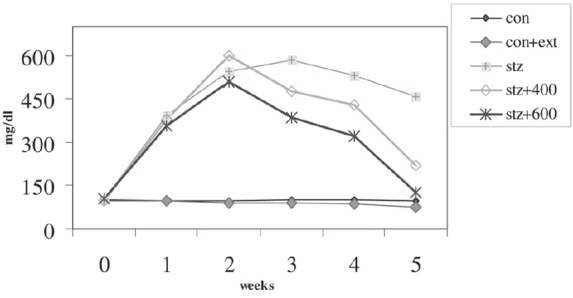
Effect of mulberyy leaves extract on blood glucose levels

The final body weight showed significant increase from the initial body weight in all the groups except in the diabetic group, in which there was significant decrease in body weight compared to the initial body weight [Table 1]. The failure of diabetic rats to gain weight during the 4-week period corresponded with the hyperglycemia seen during this period [Figure 1]. Animals of groups IV and V showed higher gain in weight as compared to those in the diabetic group but less than those in the control group.

**Table 1:** Effects of mulberry leaves extract on body weight

*Group*	*Initial weight (grams)*	*Final weight (grams)*
Group I: Control	184.2 ± 3.42^a^	204.4 ± 0.97^d^
Group II: Control mulberry	180.4 ± 6.9^a^	199.6 ± 5.38^c^
Group III: Diabetic	179 ± 3.19^a^	142.2 ± 6.41^a^
Group IV: STZ + 400	178.6 ± 6.39^a^	180.2 ± 3.27^b^
Group V: STZ + 600	174.4 ± 4.09^a^	189 ± 5.09^b,c^

The values are mean ± SE of 8 rats in each group. Means with different superscripts (a, b, c, and d) within a column are significantly different from each other at *P* < 0.05 as determined by Duncan's multiple range test

Table 2 shows the diameter of the islets and the number of β cells in all the groups. The diameter of the islets decreased in the diabetic group and the number of β cells were also significantly reduced. Both were restored after treatment with 600 mg/kg/day of extract. There was no significant change in group II either in number of β cells or in islet diameter. There was significant increase in diameter of islets and number of β cells in group IV as compared to the diabetic group.

**Table 2:** Effect of mulberry leaves extract on number of β cells and islet diameter (in micrometers) in the pancreas

*Groups*	*Control I*	*Control mulberry II*	*Diabetes III*	*Treat + 400 IV*	*Treat + 600 V*	*F value df (4,20)*

*Parameters*								
Islet diameter In μm	149.8 ± 3.91^b,c^	152.0 ± 4.32^c^	98.6 ± 4.11^a^	140.6 ± 2.36^b^	146.2 ± 2.60^b,c^	Sig *P* < 0.05
Number of β cells	172.4 ± 4.16^c^	178.0 ± 5.63^c^	115.4 ± 6.22^a^	157.2 ± 3.12^b^	168.6 ± 2.83^b,c^	Sig *P* < 0.05

The values are means ± SE of 8 rats in each group. Means with different superscripts (a, b, c, and d) within a column are significantly different from each other at *P* < 0.05 as determined by Duncan's multiple range test

Figure 1 shows the changes in fasting blood glucose level over 5 weeks. Control rats did not show any significant variation in the blood glucose throughout the experimental period. Administration of STZ (60 mg/kg) led to over 5-fold elevation of blood glucose levels, which was maintained over a period of 5 weeks. Group II animals did not vary significantly from the control group. Though *M. alba* extract, 400 mg/kg/day, reduced the hyperglycemia significantly as compared to the diabetic group, it failed to restore the level to that of the control group; with *M. alba* extract at a dose of 600 mg/kg/day, the blood glucose levels almost reached the control group level (*P* < 0.05). Thus, the ethanol extract of *M. alba* was shown to have a significant effect on blood glucose.

Table 3 shows the changes in fasting HbA1C level after 5 weeks. The ethanol extract of *M. alba* had significant effect in lowering HbA1c. After 35 days, the effect of extract on group II was not significant as compared to the control group. Treatment with extract at 400 mg/kg/day and 600 mg/kg/day decreased HbA1C significantly in the diabetic group; at 600mg/kg/day the extract lowered HbA1c to control group levels (*P* < 0.05).

**Table 3:** Effects of mulberry leaves extract on triglyceride, LDL, VLDL, HDL, cholesterol, glycosylated hemoglobin, and blood urea levels

*Group Parameters*	*Control I*	*Control mulberry II*	*Diabetic III*	*STZ + 400 IV*	*STZ + 600 V*	*F value df (4,20)*
Glycosylated hemoglobin (%Hb)	5.34 ± 0.18^a^	4.86 ± 0.08^a^	11.46 ± 0.49^c^	9.02 ± 1.07^b^	6.7 ± 0.74^a^	Sig 18.98
Triglyceride (mg/dl)	63 ± 3.13^a,b^	55.6 ± 3.12^a^	82 ± 3.78^c^	66.8 ± 2.37^b^	56 ± 2.34^a^	Sig 12.86
Cholesterol mg/dl)	62.2 ± 2.47^b^	54.6 ± 1.43^a,b^	72 ± 2.21^c^	62.6 ± 3.76^b^	47.2 ± 3.12^a^	Sig 11.82
LDL (mg/dl)	30.2 ± 1.59^b^	24.2 ± 3^a,b^	39.4 ± 2.26^c^	21.94 ± 4.7^a,b^	20.1 ± 1.28^a^	Sig 7.15
VLDL mg/dl	12.6 ± 0.82^a^	11.12 ± 1.75^a^	16.4 ± 1.06^c^	13.66 ± 2^b^	11.2 ± 0.86^a^	Sig 13.94
HDL (mg/dl)	30.4 ± 0.93^a,b^	33.2 ± 1.65^b^	27 ± 0.7^a^	29.6 ± 1.21^a,b^	31.2 ± 1.16^b^	NS 3.73
Blood urea (mg/dl)	37 ± 1.48^a^	36.4 ± 1.57^a^	77.6 ± 5.2^d^	62.4 ± 3^c^	48.2 ± 2.06^b^	Sig 31.51

The values are mean ± SE of 8 rats in each group. Means with different superscripts (a, b, c, and d) within a column are significantly different from each other at *P* < 0.05 as determined by Duncan's multiple range test

Table 3 shows the changes in fasting blood triglyceride level after 5 weeks. After 35 days, the blood triglycerides in group I and II did not differ, whereas the triglycerides were significantly elevated in the diabetic group; in group IV and V the trigyceride levels were close to the control group level (*P* < 0.05). Thus, the ethanol extract of *M. alba* had a significant effect in lowering blood triglycerides.

Table 3 shows the changes in fasting total cholesterol, LDL, VLDL, HDL, and blood urea levels after 5 weeks. Cholesterol, LDL, VLDL, and blood urea after 35 days of experiment did not differ significantly in group I and II, whereas they were elevated in diabetic group and at control group levels in group IV and V. HDL level did not alter significantly in any of the groups studied (*P* < 0.05).

## Discussion

The diminished islet size and β cell numbers resulted in the histopathology of diabetic pancreas. Reduction of β cell number and islet diameter indicates the loss of integrity between the cells in the islet. The recent report shows that the number of adult β cells can increase through self duplication/proliferation[19] supports our finding that increase in the β cell number in the diabetic islets can occur after treatment with mulberry extract. The histopathologic studies also supported our findings. STZ is believed to destroy the pancreas partially. The diabetic rats showed reduced numbers of β cells and islet diameter, but these could be restored to near normal levels by treatment with the extract of mulberry. No such changes were seen in the normal rats.

Glycohemoglobin is formed throughout the circulatory life of RBC by the addition of glucose to the N-terminal of the hemoglobin beta chain. This process, which is nonenzymatic, reflects the average exposure of hemoglobin to glucose over an extended period.

Several investigators have recommended that glycosylated hemoglobin be used as an indicator of metabolic control of diabetes since glycohemoglobin levels approach normal values in diabetics in metabolic control. In the present investigation glycosylated hemoglobin was elevated nearly 2.5 times above normal in the diabetic group. In group V, which was orally treated with 600 mg/kg/day of mulberry extract, levels of glycosylated hemoglobin approached the normal value. Andallu *et al*.,[8] in their studies on type 2 diabetic patients, administered capsules filled with powdered mulberry (at the dose of 3 g/day) and found a 10% decrease in the glycosylated hemoglobin content.

The most common lipid abnormalities in diabetes are hypertriglyceridemia and hypercholesterolemia.[20,21] Increased levels of triglycerides are a risk factor for atherosclerotic coronary disease. Repeated administration of mulberry leaf extract for 5 weeks significantly improved hypertriglyceridemia and hypercholesterolemia, bringing their levels in groups IV and V down to that of the control group.

Andallu *et al*.[8] reported a 16% decrease in triglycerides in type 2 diabetic patients after treatment with mulberry powder-filled capsules. LDL and VLDL carry cholesterol to the peripheral tissues where it is deposited; hence, high levels of LDL and VLDL are atherogenic. HDL transports cholesterol from peripheral tissues to the liver and thus aids in its excretion. HDL, therefore, has a protective effect. In the present investigation, HDL levels did not alter significantly in any of the groups. Andallu *et al*. have reported that cholesterol, LDL cholesterol, and VLDL cholesterol were reduced by 12, 23, and 17%, respectively, in type 2 diabetic patients after treatment with mulberry powder.[8] In the present investigation also, all these parameters reduced significantly in the diabetic rats and approached the levels seen in the control group.

Elevated levels of urea are seen during increased protein breakdown and may also be seen in renal disorders like glomerular nephritis and chronic nephritis. In the present investigation, elevated levels of blood urea in the diabetic group were restored to the control group level after treatment with mulberry. No earlier worker has reported similar effects on blood urea levels.

The hypoglycemic influence of mulberry leaves observed in this study concurs with the observations made by other researchers studying plant extracts. Lemus *et al.*[22] conducted short term experiments and reported hypoglycemic activity of dried levels of *Bauhinia ulrifolius*, *Galega officinalis, M. alba,* and *Rubus ulnifolius.* Sachdewa and Khemani[23] reported hypoglycemic activity of an ethanol extract of the flower of *Hibiscus rosa sinensis* on diabetes-induced rats. Andallu and Varadacharyulu[10] reported that fasting blood glucose levels in a diabetic group treated with mulberry reduced by 50%; in the present investigation, treatment with 600 mg/kg/day of mulberry extract could lower the blood glucose level to that of the control group [Figure 1]. There was no significant difference in the blood glucose levels of group I or II animals, indicating that mulberry maintains glucose homeostasis in normal conditions also.

The improvement in glycemic control, followed by the fall in VLDL production, after mulberry treatment (600mg/kg/day) could be attributed to the mulberry therapy in diabetic rats. Laakso *et al*.[24] and Laakso[25] showed improved glycemic control, followed by fall in VLDL production, in diabetic patients after treatment with oral hypoglycemic agents. The effect of mulberry on VLDL metabolism could be due to a dual mode of action: reduction in VLDL production and enhancement of VLDL removal. Earlier studies by Laakso[25] and Taskinen[26] showed higher concentration of LDL-cholesterol and lower concentration of HDL cholesterol in diabetic patients. Hypocholesteremic drugs decrease LDL-cholesterol presumably by stimulating receptor-mediated removal of LDL. This seems true of mulberry treatment also, which shows decrease in LDL but no alteration in HDL-cholesterol.

The hypoglycemic activity of mulberry leaves may be attributable to the high fiber content (13.85%) of mulberry leaves[27] and/or the presence of trigonelline bases[28] in mulberry leaves, similar to that isolated from fenugreek; it could also be due to the presence of moran A[29] and/or moranoline.[30] Mulberry leaf extract may also contain other compounds with significant hypoglycemic activity in diabetic rats.

In conclusion, the present investigation shows the therapeutic efficacy of *M. alba* leaves, at a dose of 600mg/kg/day, on a diabetes-induced experimental animal model.
